# A Composite Lactide-Mineral 3D-Printed Scaffold for Bone Repair and Regeneration

**DOI:** 10.3389/fcell.2021.654518

**Published:** 2021-07-09

**Authors:** Rayan Fairag, Li Li, Jose Luis Ramirez-GarciaLuna, M. Scott Taylor, Brian Gaerke, Michael H. Weber, Derek H. Rosenzweig, Lisbet Haglund

**Affiliations:** ^1^Department of Surgery, Division of Orthopaedic Surgery, McGill University, Montreal, QC, Canada; ^2^Research Institute of McGill University Health Center, Montreal General Hospital, Montreal, QC, Canada; ^3^Department of Orthopedic Surgery, Faculty of Medicine, King Abdulaziz University, Jeddah, Saudi Arabia; ^4^Poly-Med Inc., Anderson, SC, United States; ^5^Shriners Hospital for Children, Montreal, QC, Canada

**Keywords:** 3D printing, β-TCP, bone repair, bone substitute, scaffold, composite

## Abstract

Orthopedic tumor resection, trauma, or degenerative disease surgeries can result in large bone defects and often require bone grafting. However, standard autologous bone grafting has been associated with donor site morbidity and/or limited quantity. As an alternate, allografts with or without metallic or polyether-etherketone have been used as grafting substitutes. However, these may have drawbacks as well, including stress shielding, pseudarthrosis, disease-transmission, and infection. There is therefore a need for alternative bone substitutes, such as the use of mechanically compliant three-dimensional (3D)-printed scaffolds. Several off-the-shelf materials are available for low-cost fused deposition 3D printing such as polylactic acid (PLA) and polycaprolactone (PCL). We have previously described the feasibility of 3D-printed PLA scaffolds to support cell activity and extracellular matrix deposition. In this study, we investigate two medical-grade filaments consistent with specifications found in American Society for Testing and Materials (ASTM) standard for semi-crystalline polylactide polymers for surgical implants, a pure polymer (100M) and a copolymeric material (7415) for their cytocompatibility and suitability in bone tissue engineering. Moreover, we assessed the impact on osteo-inductive properties with the addition of beta-tricalcium phosphate (β-TCP) minerals and assessed their mechanical properties. 100M and 7415 scaffolds with the additive β-TCP demonstrated superior mesenchymal stem cells (MSCs) differentiation detected *via* increased alkaline phosphatase activity (6-fold and 1.5-fold, respectively) and mineralized matrix deposition (14-fold and 5-fold, respectively) *in vitro*. Furthermore, we evaluated *in vivo* compatibility, biosafety and bone repair potential in a rat femur window defect model. 100M^+β^^-*TCP*^ implants displayed a positive biosafety profile and showed significantly enhanced new bone formation compared to 100M implants evidenced by μCT (39 versus 25% bone volume/tissue volume ratio) and histological analysis 6 weeks post-implantation. These scaffolds are encouraging composite biomaterials for repairing bone applications with a great potential for clinical translation. Further analyses are required with appropriate evaluation in a larger critical-sized defect animal model with long-term follow-up.

## Introduction

Currently, bone is one of the most transplanted types of tissues (Brantigan and Steffee, 1993; Gómez et al., 2016) with more than 2 million bone grafting procedures annually in different surgical fields (Giannoudis et al., 2005). Furthermore, the repair of large bone defects resulting from tumor resection, major traumatic injuries, debridement of infected, defective or degenerate tissue is a challenging surgical problem (Zhang et al., 2016). For long bones and spine reconstruction, metallic implants with allografts show positive short-term results, have high mechanical strength and reduce the need for autografts. However, some are unfortunately associated with long-term complications such as fibrocartilage formation, poor mineralization, and overall failure of osseointegration (Togawa et al., 2004). While the FDA has recently approved the use of 3D printed titanium spinal cages, metallic implants have classically been non-degradable and produce a significant mismatch with the mechanical properties of bone, leading to a stress shielding phenomena, corrosion, wear, and ultimately implant migration (McAfee et al., 1989, McAfee et al., 1999; van Dijk et al., 2002b). Although there is emergence of magnesium based degradable implants, current metallic implants are considered as permanent foreign bodies within the host, which may eventually require a secondary removal intervention (Glassman et al., 1996). Due to such shortcomings, there is a need to develop new innovative materials which possess mechanical strength, are bioresorbable and promote bone regeneration with better understanding for bone healing and clinical applicability.

Three-dimensional-printing is a layer-by-layer method to manufacture physical models from a computer-aided design virtual model. It allows for the rapid generation of scaffolds with complex geometry and mechanical properties using a variety of materials conducive to biomedical applications (Ahangar et al., 2019a,b; Haglund et al., 2019). Three-dimensional-printed polylactic acid (PLA) or polycaprolactone (PCL) scaffolds can be fabricated and structured as customized implantable scaffolds for spine or long bone reconstruction. Indeed, PCL scaffolds have been shown to promote the complete repair segmental critical sized tibial defects in sheep (Reichert et al., 2012; Cipitria et al., 2013). PCL and PLA scaffolds are generally recognized as safe materials by the FDA, and their mechanical properties can be comparable to those of trabecular bone (Haglund et al., 2019). They are also biodegradable, biocompatible and relatively inexpensive compared to costs associated with metal additive manufacturing (Ni et al., 2019). Newly growing and remodeling bone can easily replace the degrading polymer over time, which eliminates the risk for secondary surgeries for foreign body removal. These types of polymeric scaffolds can also be used as drug releasing systems (Wuisman and Smit, 2006; Ahangar et al., 2018; Akoury et al., 2019). However, their natural composition and the resorption rate should be taken into consideration to prevent abnormal tissue response (Bostman et al., 1990). Therefore, a careful choice of material with the appropriate degradation and neo-bone formation rate, suitable structural morphology, and optimal porosity and pore size for sufficient vascularization is of utmost importance (Bergsma et al., 1995; Haglund et al., 2019). Many biodegradable lactide-based implants have been developed as orthopedic grafts, screws, and plates (Kulkarni, 1966; Kulkarni et al., 1971; Bostman et al., 1990; Bergsma et al., 1995), and clinical trials have shown favorable initial results (Bostman et al., 1987; Hope et al., 1991; Casteleyn et al., 1992; Korhonen et al., 2018). However, a lack of long-term follow-up, small sample size of the trials and failure to prove the safety and advantages are considered limitations of these studies. Such polymers have been applied in loaded bones and spine surgeries such as fixation pins (Deguchi et al., 1998), joint fixation rods (Johnsson et al., 1997), and stabilization devices (van Dijk et al., 2002c, 2005; Vaccaro et al., 2003; Wuisman and Smit, 2006). The inconclusive comparisons between these studies along with the focus on non-loaded bone defects such as craniofacial defects have had negative impact on their use as alternatives to pure metallic devices in mechanically loaded bones.

Three-dimensional printable materials such as PLA, PCL and poly lactic-co-glycolic acid (PLGA) have demonstrated encouraging outcomes for tissue engineering *in vitro*. However, optimal and mainstream use *in vivo* has yet to be achieved as evidenced by longer degradation periods, unwarranted inflammatory responses, and sub-optimal mechanical properties (Okada, 2002; Sabir et al., 2009). Our group and others have previously applied commercially available extrusion-based fused deposition 3D printing to design and fabricate scaffolds suitable for bone, intervertebral disc tissue engineering, and therapeutic delivery strategies (Rosenzweig et al., 2015; Akoury et al., 2019; Fairag et al., 2019; Li P. et al., 2019; Velioglu et al., 2019; Cooke et al., 2020; Korn et al., 2020). We have previously identified a 750 μm optimal pore size for 3D printed PLA scaffolds which support mesenchymal stem cell (MSC) proliferation and calcified matrix generation (Fairag et al., 2019). We demonstrated that this approach can be easily modified and enhanced to improve structural and functional properties. Furthermore, composites of similar materials including blends of mineral such as tricalcium phosphates or hydroxyapatite have shown to have improved pro-osteogenic features, good mechanical strength and *in vivo* compatibility (Bruyas et al., 2018; Haglund et al., 2019; Li X. et al., 2019).

In this study, we generated 3D printed scaffolds with previously optimized 750 μm pore size (Fairag et al., 2019) using two novel medical-grade, commercially produced materials composed of pure lactide (100M) and copolymer of lactide, trimethylene carbonate (TMC) and caprolactone (7415). Each material was evaluated with or without commercially blended beta-tricalcium phosphate (β-TCP). To the best of our knowledge, these materials represent one of two companies producing such “ready-to-print” materials – the other being 20% β-TCP PCL filament from Biomaterials USA (not confirmed to be medical grade). We assessed their mechanical and wettability properties and their potential to induce MSCs osteogenic differentiation and bone-like matrix production. We further evaluate the scaffolds *in vivo* in a femoral window defect primarily for biocompatibility and biosafety (Prakasam et al., 2017; Ida et al., 2018). Secondarily, we assessed the potential for bone repair in this pilot study. The results here demonstrate that our scaffolds may be useful as bone graft or interbody cage alternatives, warranting future extensive *in vivo* assessment in large bone defects.

## Materials and Methods

### Material Composition and Fabrication

Filaments (Lactoprene^®^ 100M and Lactoprene^®^ 100M^+β^^-*TCP*^, Lactoprene^®^ 7415, and Lactoprene^®^ 7415^+β^^-*TCP*^) were kindly provided by Poly-Med, Inc. (Anderson, SC, United States) with 1.75 mm diameter. All molecular weights are listed in Table 1. The Lactoprene 100M group are composed of 100% lactide [linear homopolymer with medium viscosity as determined with gas chromatography (GC) and compared to the 7415 polymer (Table 1)] whereas the Lactoprene 7415 group are composed of 74% Lactide, 15% Trimethylene Carbonate, 11% Caprolactone (polyaxial block copolymer). Lactoprene 100M^+β^^-*TCP*^ and Lactoprene 7415^+β^^-*TCP*^ polymers included a process of blending of 30 weight% β-TCP particles (with a mean diameter of less than 5 μm) dispersed throughout to the original standard filaments. Scaffolds with 750 μm pore size were printed as described previously (Fairag et al., 2019) using Flashforge Creator Pro 3D Desktop Printer (Flashforge, Los Angeles, CA, United States) with a 0.3 mm nozzle. The printing speed (18 mm/s) was the same for all scaffolds except the 7415^+β^^-*TCP*^ group (the speed was reduced by 10% after printing half of the construct). The print temperature for 100M was 190°C, and the temperature for 7415 was 220°C. Following printing, the constructs were packaged, taped and autoclaving was performed for 15 min at 121°C under 15 psi pressure. We have previously reported that this method sterilizes the scaffolds without compromising functionality (Fairag et al., 2019).

**TABLE 1:** Composite profiling.

Material	DSC					GPC			IV, dL/g	GC
	T_*g*_, °C	T_*c*_, °C	ΔH_*c*_, J/g	T_*m*_, °C	ΔH_*f*_, J/g	M_*n*_, da	M_*w*_, da	PDI		Total residual monomer, %
100M	70.2	118.7	32.8	182.6	32.9	110,139	189,981	1.72	1.59	0.20
100M^+β^^-*TCP*^	70.6	112.0	22.9	182.6	29.0	101,005	155,933	1.54	1.24	0.05
7415	No peak	90.9	6.5	165.9	23.2	27,816	65,133	2.34	0.58	0.09
7415^+β^^-*TCP*^	51.5	88.0	7.0	165.9	14.7	61,638	114,838	1.86	0.96	0.32

*The molar composition, molecular weights, and thermal properties of the prepared 3D-printed scaffolds. DSC, differential scanning calorimetry; T_*g*,_ glass transition temperature; T_*c*_, peak of the crystallization endotherm; ΔH_*c*,_ heat of crystallization; T_*m*_, peak of the melting endotherm; ΔH_*f*_, heat of fusion; GPC, gel permeation chromatography; M_*n*_, number average molecular weight; M_*w*_, weight average molecular weight; PDI, polydispersity index; IV, inherent viscosity; GC, gas chromatographic technique.*

### Composites Characterization

To assess the materials characterization after 3D printing and to determine their physical and chemical properties, molecular weights were characterized using two methods. First, gel permeation chromatography (GPC, Waters Corporation, Milford, MA, United States) using dichloromethane as a mobile phase and polystyrene standards was utilized to determine the weight average molecular weight (M_*w*_), number average molecular weight (M_*n*_), and polydispersity index (PDI), which is the ratio of M_*w*_/M_*n*_ used to indicate the distribution of molecular weight within a sample. Dilute solution inherent viscosity (IV) was determined using a Cannon-Fenske U-viscometer at 20°C at a concentration of 0.1 mg/mL in chloroform. In cases of materials containing β-TCP, samples were first separated to remove ceramic particulate and isolate the polymer being analyzed.

Thermal profiles were evaluated by differential scanning calorimetry (DSC) using a Perkin Elmer Pyris DSC (Waltham, MA, United States). Samples of 4–10 mg were weighed and placed in hermetically sealed aluminum pans and heated from 20 to 240°C at a heating rate of 20°C/min. Thermograms were analyzed for glass transition temperature (T_*g*_), peak melting temperature (T_*m*_) and heat of fusion (ΔH_*f*_), all of which were calculated directly using the onboard software.

Gas chromatography was performed on a Claris 580 system (Perkin Elmer, Waltham, MA, United States) equipped with a flame ionization detector, using helium as the carrier gas, and used to quantify the level of residual monomer using a direct injection technique. Samples were first dissolved in hexafluoro-2-propanol (HFIP) solvent and directly injected into the column. Individual peaks were matched with retention times and calibration curves of matching monomer standards (lactide, caprolactone, and TMC) to determine individual and sum total monomer residuals in the dissolved sample. In cases of materials containing β-TCP, samples were first separated to remove ceramic particulate and isolate the polymer being analyzed.

### Surface Characterization

Measuring the wettability of the material surface and how the scaffold interact with the cells is an important parameter in understanding the hydrophilic/hydrophobic characteristics of our constructs. A contact angle below 90° defines the material as hydrophilic while a contact angle higher than 90° resembles a hydrophobic surface. Small monolayer circular plates were designed and printed with all materials for this test to exclude porous orthogonal structural effects. Sessile contact angle measurements were calculated using an OCA 15EC measuring device (Data Physics, San Jose, CA, United States) with a dosing volume of 4 μl of 37°C deionized water with a rate of 0.5 μl/s dispensed through a 500 μl Hamilton syringe onto the top surface of each scaffold (*n* = 3). The drop shape was documented using a fixed high-speed framing camera. Measurements were calculated at the time the droplet attaches to the scaffold’s surface and after 30 s.

### Degradation Profile

Scaffolds were weighed and submerged in 3 mL of 0.05M HCl–Tris buffer solution with a pH of 7.4 (all placed in a 5 mL Eppendorf tube) simulating the biodegradation process *in vivo* under the same incubation condition for 21 days dynamically on a nutator at 37°C (*n* = 3). After the incubation period, scaffolds were allowed to dry, and the weight loss was calculated as following:

$$\text{Degradation}=\left(W_{1}-W_{2}\right)/W_{1}\times 100\%$$

In which (*W*_1_) refers to the initial dry weight of samples and (*W*_2_) refers to the dry weight of samples after incubation periods.

### Mechanical Properties

Unconfined axial compression was applied to dry freshly printed scaffolds (*n* = 3) at a rate of 0.1 mm/s until reaching the failing point using a Mini Bionix 858 (MTS machine) as previously described (Fairag et al., 2019). Young’s modulus was calculated using the slope of the stress-strain curve according to the surface area of each scaffold.

### Cell Seeding on Scaffolds

Human bone marrow derived MSCs (Rooster Bio Inc., Frederick, MD, United States) were cultured in a 5% CO_2_ incubator at 37°C using High glucose-Dulbecco’s Modified Eagle Medium (DMEM) supplemented with 10% fetal bovine serum (FBS) (Gibco, Burlington, ON, Canada), 1% glutaMAX supplement and 0.5% gentamycin. When the cells reached 80% confluency, they were trypsinized and subcultured. Our previously described “Syringe technique” was used to seed a 5 × 10^5^ cell/scaffold (Fairag et al., 2019). Cells from the third passage were seeded into scaffolds in the following experiments. After 24 h of seeding, scaffolds were moved to a new 24-well plate and supplied with either standard (STD) or osteogenic (OST) media, where the standard media was composed of (DMEM high glucose, 10% FBS, and 1% gentamycin), and (DMEM low glucose, 10% FBS, 1% gentamycin, 50 μg/ml ascorbic acid, 10 nM dexamethasone, and 5 mM betaglycerol-2-phosphate) was used for the osteogenic differentiation media. Medium was changed every 3–4 days.

### Cell Proliferation Within Scaffolds

A total of 21 days after seeding into scaffolds, the cell numbers on each scaffold were determined by DNA assay as previously reported (Hoemann, 2004). Briefly, cell-seeded scaffolds were incubated in 1 mL of 4M guanidine hydrochloride (GuHCl) buffer supplemented with complete protease inhibitor cocktail (Roche Applied Science, Indianapolis, IN, United States) for 48 h in a shaking bath at 4°C. The resulting mixture was centrifuged and aliquots (20 μl) of the supernatants were diluted with 1X TNE buffer (10 mM TRIS–HCl, 50 mM NaCl, 1 mM EDTA, pH 8.0) to fit into the standard curve and mixed with (90 μL) of Hoechst 33258 working solution (100 ng/ml, Thermo Fisher Scientific). DNA content was quantified spectro-fluorometrically using a T-Can multi-mode detection reader at a wavelength of 352 nm (emission wavelength of 461 nm) by correlating with a DNA standard curve which was generated by serial dilutions of calf thymus DNA (10 mg/ml). Adding equal volumes of TNE and Hoechst 33258 dye working solution was used as blank controls which were then subtracted from the corresponding samples. Detached cells after 24 h of seeding were collected and the DNA was quantified and subtracted from the DNA content of 5 × 10^5^ cells and expressed as (attachment ratio).

### Cell Distribution Within the Scaffolds

The distribution of the cells in the constructs cultured under the osteogenic induction condition for 1 week were observed by confocal microscopy (Zeiss LSM780). Briefly, cells were labeled with red fluorescent membrane dye (Vybrant^TM^ Cell labeling solutions, Thermo Fisher Scientific) during the seeding process for visualization. Empty scaffolds were also visualized as negative controls to avoid scaffold background effects. To analyze the distribution of cells in the scaffolds, the volume data were used to create 3D renderings and Z-stacks of the cell-seeded scaffolds using Zeiss Zen software (Carl Zeiss, Oberkochen, Germany).

### Scanning Electron Microscopy Observation

Constructs of all four materials were visualized by scanning electron microscopy (FEI Inspect F50 FE-SEM) after a full culture period. Briefly, scaffolds were fixed with 4% paraformaldehyde and subsequently underwent a drying process and were coated with platinum sputter as previously described (Kemmenoe and Bullock, 1983). Empty scaffolds were also scanned as controls. Morphological characteristics of the constructs, infused β-TCP minerals and attached cells were imaged using SEM parameters of low voltage 5 kV on high resolution setting. X-ray photoelectron spectroscopy (XPS) was conducted to identify and verify the presence of calcium phosphate particles on the surface of Lactoprene 100M^+β^^-*TCP*^ and Lactoprene 7415^+β^^-*TCP*^ constructs after printing.

### Calcified Matrix Mineralization

Alizarin Red staining was used to determine the extracellular matrix mineralization. Following culture, MSCs seeded scaffolds (STD and OST) and empty controls that cultured in osteogenic differentiation media for the same period were fixed with 4% buffered paraformaldehyde solution (PFA), then stained with 1% Alizarin Red solution (Sigma-Aldrich Inc., Darmstadt, Germany) for 10 min. Excess stain was removed, and scaffolds washed gently with deionized water and dried. Alizarin red stain was dissolved in 10% acetic acid, processed, and subsequently quantified using the osteogenesis assay kit (ECM815 Millipore Sigma Inc., Canada) according to the user manual. The optical density of the absorbance at 405 nm was measured. Values were corrected to the empty controls and normalized to STD culture values. We also quantified alkaline phosphatase (ALP) activity using colorimetric kinetic QuantiChrom^TM^ assay (VWR scientific, Canada) according to the manual’s protocol. Briefly, 50 μl of samples were mixed with 150 μl of working solution composed of fresh reconstituted assay Buffer, 5 mM Mg Acetate and 10 mM p-nitrophenyl phosphate liquid substrate. Readings were measured at (OD405 nm) at the start, and again after 4 min. All quantification and calculations were performed according to the manufacturer’s specifications and instructions.

### *In vivo* Experimental Design

We assessed the biosafety and the bone regeneration capacity of 100M and 100M^+β^^-*TCP*^ implantable scaffolds within a non-critical sized defect in rat femora. Ethical approval was obtained from the local institutional ethics committee (2018-8038). Surgeries were performed at (RI-MUHC, ARD Center, Montreal QC, Canada) under aseptic conditions. Total of 16 male Sprague Dawley (Charles River Laboratories) aged (13–15 months) weighted (550–600 g) were assigned to this protocol. Rats were given Buprenorphine Slow Release (1 mg/kg, one dose subcutaneously 30 min Pre-op for 72 h), Carprofen (5 mg/kg subcutaneously 30 min Pre-op, and Q24h for 3 days) for pain control. Rats were anesthetized in an induction chamber with 5% isoflurane (Baxter, USP), 1.5 L/min oxygen. Eye ointment was applied, and the rats were transferred to vaporizer mask and anesthesia was adjusted. Surgical area was shaved and disinfected. Rats then positioned in a lateral recumbency, placed on a heated pad (37°) on sterile table and covered with sterile drape with only the surgical area exposed. An approximately 3 cm long skin incision was made midway between the greater trochanter (hip) and the lateral condyle (knee), and the femur was exposed in between the biceps femoris and vastus lateralis muscles. Muscles were retracted using small retractors for full exposure of the bone before drilling. A unicortical defect (6 × 2 mm) was created carefully using a low-speed drill (Micro Drill, USA power cord 75-0900) with spherical drill bur size (1.9 mm). Defect was washed with normal saline and packed with Small specks of absorbable hemostatic Gelatin Sponges (Johnson & Johnson, Medical, New Brunswick, NJ, United States) to stop bleeding. All steps were performed under continuous sterile saline irrigation to avoid thermal insult and soft tissue injury. Defects were randomly fitted with either 100M or 100M^+β^^-*TCP*^ sterile 3D-printed scaffold. Following this, retractors were removed, and fascia was sutured with 6.0 Vicryl sutures (Ethicon, Norderstedt, Germany), and the skin incision is closed with 4.0 Prolene sutures (Ethicon, Norderstedt, Germany). The same surgical procedure was performed on other side leg. The rats were kept in solitary cages with courses of post-surgical analgesics, monitored daily for signs of pain and irritability. Blood was collected twice during the experiment (1-day pre-op as a baseline, 6-weeks post-op) and analyzed (Diagnostic Laboratories, CMARC, McGill University). X-ray of the limbs were obtained after the surgery to confirm the positioning of the scaffold, intact opposite cortex and to rule out fractures at site of operation and 6-weeks post-op. At euthanasia, the femora were disarticulated at the knee and the hip and whole femora were collected.

### μCT Analysis

Rat femora with inserted scaffolds were carefully dissected free of soft tissue, fixed for 24 h in 4% paraformaldehyde at 4°C and rinsed thoroughly with sterile PBS prior to micro-computed tomography (μCT) analysis. Scans were performed at 9 μm/pixel resolution on a Skyscan 1172 instrument (Bruker, Kontich, Belgium) using a 1.0 mm aluminum filter at a voltage of 59 kV, a current of 167 μA. The scan projections were reconstructed into 3D models using NRecon software v.1.6.10.4 (Bruker) and loaded into CTAn software v.1.16.4.1 (Bruker) for analysis. A region of interest (ROI) measuring 4 mm long × 2 mm wide × 2 mm deep was created in the middle of the bone window defect and encompassing the defect, scaffold and medullary cavity of the femur. Quantitative data for mineralized tissue includes bone quantity (BV/TV), number (Tb.N), thickness (Tb.Th), and separation of trabeculae (Tb.Sp), connective density (Conn.Dn), total porosity (Po.Tot), and number of closed pores (Po.N.cl, corresponding to osteocyte lacunae), as described (Ramirez-Garcia-Luna et al., 2019).

### Histological Assessment

At 6 weeks after implantation, the animals were scanned with X-ray prior to euthanasia, and the bilateral femora were collected. Following fixation and μCT scanning the samples were then dehydrated with ethanol gradient (70, 90, and 100%, respectively) and processed for polymethylmethacrylate (PMMA) embedding (Henderson et al., 2011). Following embedding, samples were sectioned using a microtome (Microm HM 355S, Microm International GmbH, Walldorf, Germany) at 6 μm thick and subsequently stained with von Kossa and counter stained with toluidine blue (Sigma-Aldrich Inc., Darmstadt, Germany) to assess mineralized tissue and with paragon multiple stain solution (MSS) for basic histological assessments, as previously described (Ramirez-GarciaLuna et al., 2017). Microscopic images were captured with a Zeiss Axioskop 40 microscope (Carl Zeiss, Toronto, ON, Canada).

### Statistical Analysis

All values are stated as mean ± standard deviation (SD). At least three independent experiments (three replicates) are represented in each graph. Analysis of variance (ANOVA) with appropriate corrections for multiple comparisons were used for analyzing the data. μCT analysis was carried out using the R v.3.4.1 (The R Core Team, 2018) statistical software. Shapiro–Wilk tests were used to determine the normal distribution of the data, and after confirming it, ANOVA was used to assess the differences between groups. *P*-values of (<0.05) were considered significant. GraphPad Prism version 6.0 was used for all other statistical analyses (GraphPad Inc., La Jolla, CA, United States).

## Results

### Scaffold Fabrication and Material Profiling

**FIGURE 1:**
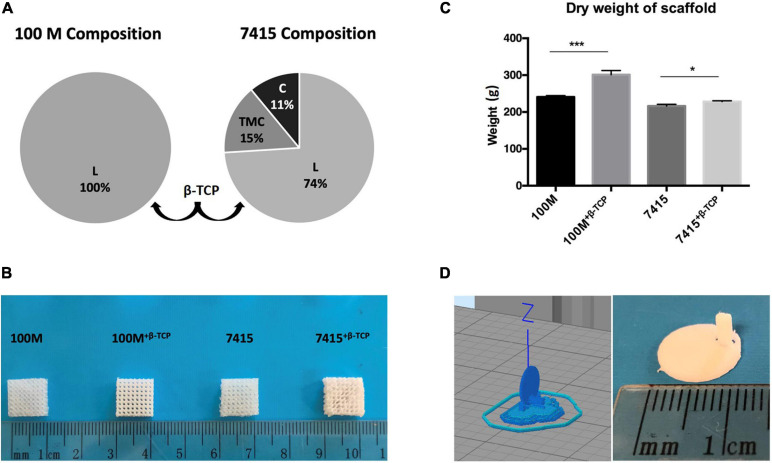
Scaffold fabrication. **(A)** Illustrated charts representing the total composition of 100M scaffolds and 7415 scaffolds. **(B)** Scaffolds professional images. Marker represent 1 cm dimensions. **(C)** Graph showing the differences in the dry weights of materials after printing, *n* = 3, error bars represent ±SD, and *P-value < 0.05, ***P-value < 0.001. **(D)** Circular scaffold design for testing surface characteristics of materials.

The two scaffold composition designs (Figure 1A) produced identical printed scaffolds with uniform pore distribution and dimensions (Figure 1B; Fairag et al., 2019). The weight of each structure was recorded after printing. It is clear that the addition of β-TCP particles had a significant impact on weight (100M: 240.6 mg ± 1.5 SD, 100M^+β^^-*TCP*^: 301 mg ± 4.5 SD, 7415: 216 mg ± 2 SD, 7415^+β^^-*TCP*^: 228 mg ± 0.8 SD) (*P*-value < 0.05 to <0.0001) (Figure 1C).

In order to evaluate the changes in material properties as a consequence of thermal exposure during 3D printing, DSC, GPC, and GC analysis were conducted including the molar composition, molecular weights, and thermal properties of 3D-printed scaffolds (Table 1). The endothermic peaks at 182.6 and 165.9°C are reflective of the melting points of 100M and 7415. The presence of β-TCP did not substantially influence T_*g*_ and T_*m*_ of the material. All materials possessed a very low degree of crystallinity of about 0.2% for 100M, 0.05% for 100M^+β^^-*TCP*^, 0.09% for 7415 and 0.32% for 7415^+β^^-*TCP*^.

### Scaffold Surface Morphology

**FIGURE 2:**
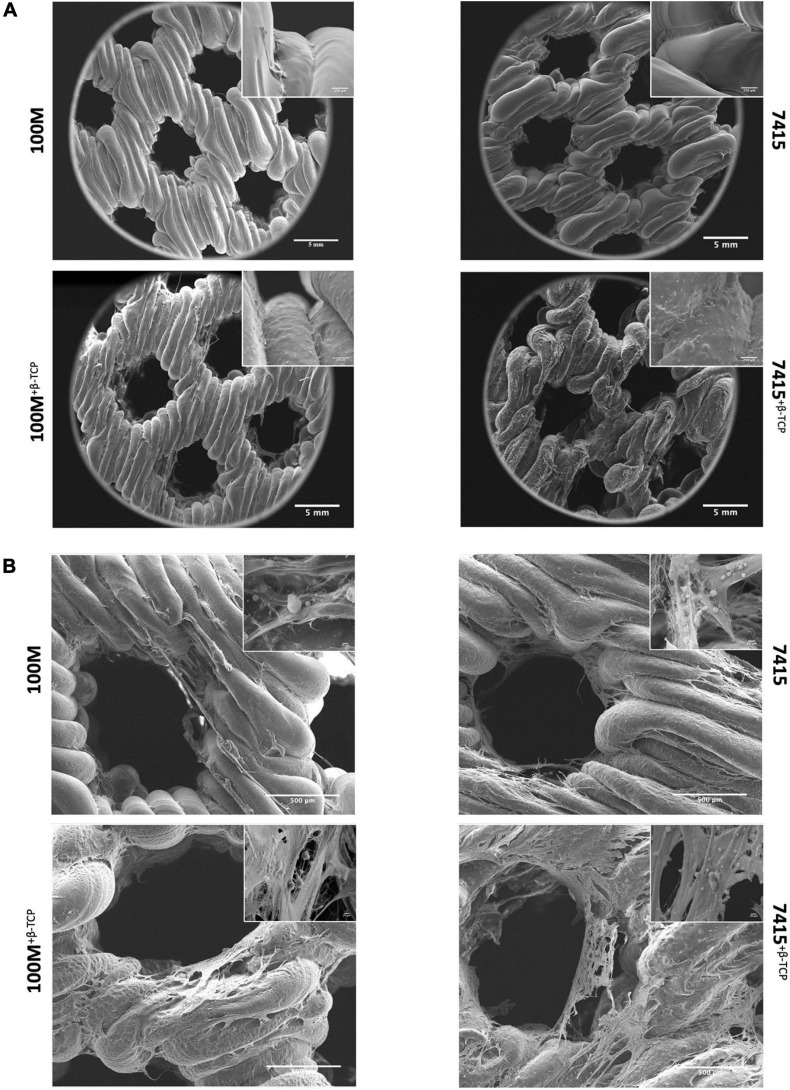
Surface morphology of scaffolds with and without cells. **(A)** Scanning electron microscopy of empty scaffolds. Representative SEM images at 80× and 1500× magnifications and scale bars represents 5 mm, 250 μm (*n* = 3). **(B)** Cell-seeded scaffolds were clearly covered with matrix deposition after 21 days of culture at 200× and 6000×x magnifications and scale bars represents 500, 20 μm (*n* = 3).

**FIGURE 3:**
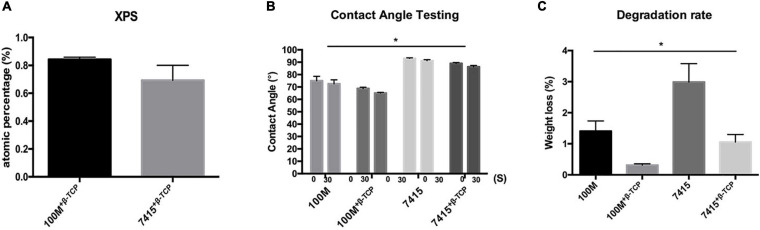
Scaffold characterization. **(A)** X-ray photoelectron spectroscopy graph of 100M^+β^^-*TCP*^ and 7415^+β^^-*TCP*^ showing the surface atomic percentage of calcium phosphate normalized to 100M and 7415, respectively. **(B)** Contact Angle test represents surface wettability of the materials at 0 s and after 30 s. *n* = 3, error bars represent ±SD, and *P-value < 0.05. **(C)** Degradation profile of all materials represents the percentage of weight loss after 21 days of soaking in Tris–HCl. *n* = 3, error bars represent ±SD, and *P-value < 0.05.

Surface morphologies of the samples were assessed by SEM (Figure 2A). Scaffolds without β-TCP mineral displayed a smooth surface, while the scaffolds with β-TCP showed a rougher surface. Inset images further show that β-TCP particles were distributed evenly and all over the scaffold surfaces. XPS quantification of the atomic presence of calcium phosphate on the surface of each material was performed. 100M^+β^^-*TCP*^ displayed 0.84 ± 0.01% SD and 7415^+β^^-*TCP*^ had 0.62 ± 0.03% SD. However, 100M and 7415 scaffolds showed no presence of calcium phosphate on their surfaces (Figure 3A).

### Contact Angle Measurement

Based on the structure design shown in Figure 1D, the apparent contact angle demonstrating the water droplet form at different time points (0 and 30 s) is shown in Figure 3B. The graph indicates that the contact angle of all materials was reduced with time suggesting that materials are absorbing the dispensed liquid. A statistical difference between materials demonstrated wettability which can be reflected in cytocompatibility and degradation profile. All materials recorded contact angles of 90° or less, indicating hydrophilic characterization. Multiple comparisons show significant differences between materials (100M: 75° ± 3.5° SD at 0 s and 72.6° ± 3.1° SD at 30 s, 100M^+β^^-*TCP*^: 68.8° ± 0.98° SD at 0 s and 65.1° ± 0.55° SD at 30 s, 7415: 93.3° ± 0.66° SD at 0 s and 91.55° ± 0.98° at 30 s, 7415^+β^^-*TCP*^: 82.2° ± 1.1° SD at 0 s and 70.3° ± 1.9° SD at 30 s).

### Degradation Properties

Degradation behavior of these novel materials is an important factor affecting their performance and behavior when further implanted *in vivo* especially during the early stages of fracture healing when withstanding applied mechanical loads is needed. Accurate evaluation of the degradation profile of synthetic polymers can take up to several months, thus *in vitro* induced accelerated degradation using alkaline medium reflects in a short-term the hydrolysis process similar to the *in vivo* conditions. The weight loss of all materials in Tris–HCl buffer at pH 7.6 is shown in Figure 3C. After being soaked for 21 days, the weight loss between all the four groups showed a statistical difference (*P*-value < 0.05). All the samples showed weight loss by 21 days (100M: 1.4 ± 0.32% SD, 100M^+β^^-*TCP*^: 0.31 ± 0.045% SD, 7415: 2.98 ± 0.59% SD, 7415^+β^^-*TCP*^: 1 ± 0.24% SD). Scaffolds containing β-TCP minerals showed a significantly slower degradation rate. Ultimately, the 7415 scaffolds became fragmented into small pieces. The above results revealed that the incorporation of β-TCP minerals significantly slows the degradation rate, which may further contribute to their important role for bone tissue engineering.

### Mechanical Stiffness

**FIGURE 4:**
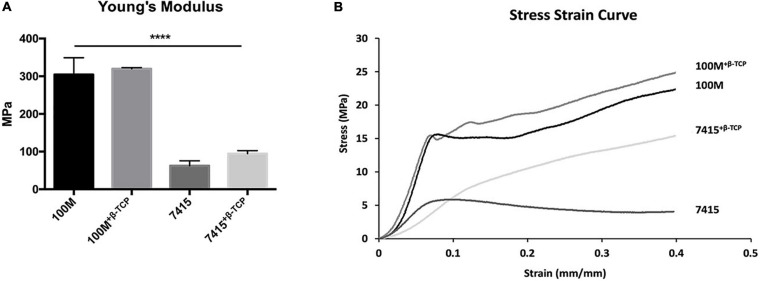
Mechanical properties of novel 3D printed scaffolds. **(A)** Young’s modulus representing 5–10% compressive stress/strain curves of printed, acellular scaffolds. For each set, *n* = 3, error bars represent ±SD (*P*-value < 0.0001). **(B)** Representative stress/strain curves of all materials showing the amount of deformation, elastic (proportionality) limit and plastic region. For each set, *n* = 3. ****p < 0.0001.

The compressive mechanical properties of scaffolds with different materials are presented in Figure 4A. Significant differences in stiffness were observed between the 100M group and the 7415 group (*P*-value < 0.0001). The Young’s Modulus obtained from the linear slope for the 100M was (304.56 MPa ± 44.69 SD), 100M^+β^^-*TCP*^ scaffold (319.63 MPa ± 2.77 SD), 7415 scaffolds (62.56 MPa ± 12.95 SD) and (94.5 MPa ± 7.90 SD) for 7415^+β^^-*TCP*^ scaffolds (Figure 4B). There was also a statistical difference between the two groups containing β-TCP, clearly indicating that 100M^+β^^-*TCP*^ is the stiffest material among the groups, and similar in strength to 100M. This makes them more suitable for bone tissue engineering as they showed much better compressive properties than those of the PLA scaffolds we described previously (Fairag et al., 2019). The 7415-group showed low mechanical characteristics which favor them in minimally supporting applications such as craniofacial repair.

### Cell Morphology, Attachment, and Proliferation

**FIGURE 5:**
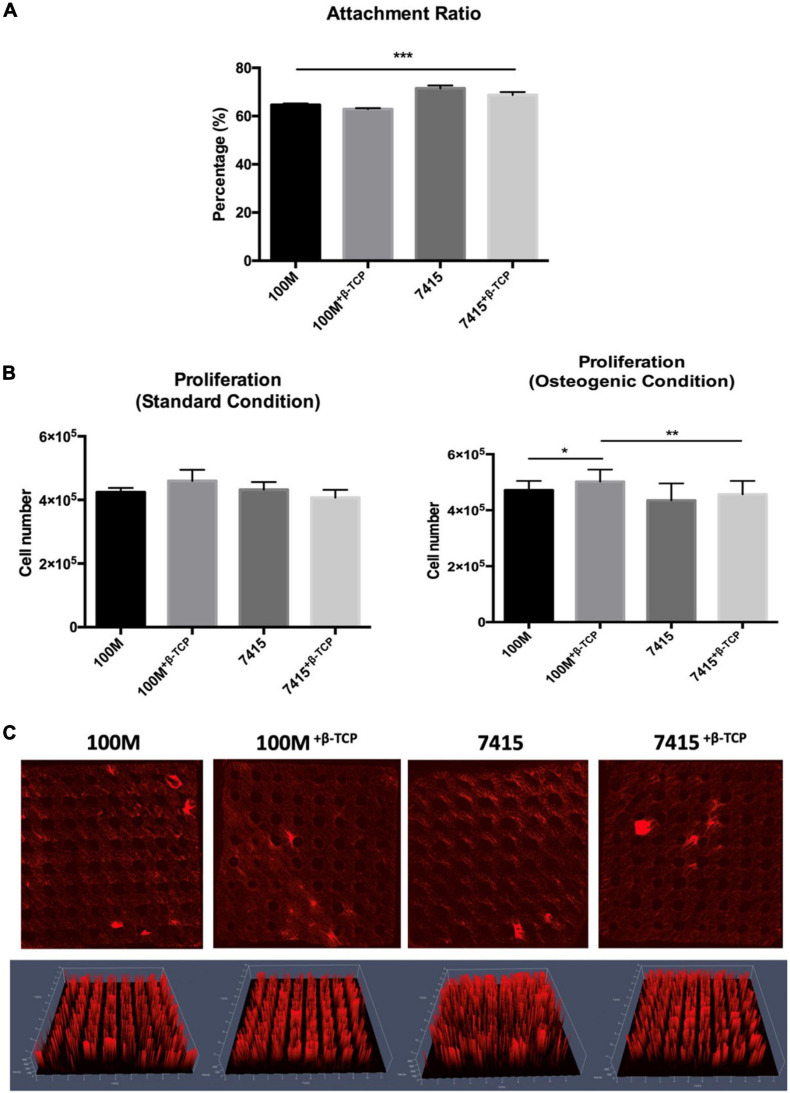
Quantification of DNA and cell distribution. **(A)** Graph represents the attachment ratio quantifications of cell-seeded into each scaffold (*n* = 3 in triplicate), error bars represent ±SD, and ***P-value < 0.001. **(B)** Proliferation graph present number of cells in each scaffold after 21 days in standard and osteogenic culture (*n* = 3), error bars represent ±SD, and *P-value < 0.05, **P-value < 0.01, ***P-value < 0.001. **(C)** Representative maximum intensity projection images showing scaffolds seeded with labeled cells after 1 week in osteogenic culture using confocal microscopy. Accordingly, 2.5D rendering images using (Zeiss Zen software) presenting the middle of the scaffold (2 mm *Z*-axis) (*n* = 3).

Scanning electron microscopy observation revealed that cells are covering the scaffolds in the inner and outer surfaces, forming interconnections, and clusters coating the surface of the scaffolds (Figure 2B). Cells had rounded or polygonal shapes with multiple interconnections forming a web-like matrix net within the pores. Distinctive β-TCP particles were identified within appropriate scaffolds. The number of cells initially adhering to the 7415 group was significantly higher than that of 100M groups scaffolds (100M: 64.66% ± 0.59% SD, 100M^+β^^-*TCP*^: 62.89% ± 0.43% SD, 7415: 71.53% ± 1.15% SD, 7415^+β^^-*TCP*^: 68.74% ± 1.26% SD) (*P*-value < 0.0001) (Figure 5A). Despite that, all materials supported high cell proliferation and showed an increase in cell numbers after the culture period. Eventually, the 100M group scaffolds recorded higher cell numbers under both standard and osteogenic conditions compared to the 7415 group in which 100M-STD had approximately 4.25 × 10^5^ cells ± 13,516 SD and 100M OST had 4.7 × 10^5^ cells ± 32,948 SD, whereas 100M^+β^^-*TCP*^-STD scaffolds had around 4.6 × 10^5^ cells ± 35,422 SD, and 100M^+β^^-*TCP*^-OST had more than 5 × 10^5^ cells ± 42,612 SD. The 7415 scaffolds showed 4.3 × 10^5^ cells ± 23,849 SD and 4.3 × 10^5^ cells ± 60,497 SD under both standard and osteogenic conditions, respectively. The 7415^+β^^-*TCP*^ had 4.0 × 10^5^ cells ± 24,330 SD under the standard condition and 4.5 × 10^5^ cells ± 47,991 SD under the osteogenic condition (Figure 5B).

### Cell Distribution on Scaffolds

One week after seeding cells into scaffolds, confocal microscopy revealed that the cells of all the scaffolds have firmly attached to the surface and micropores, they are covering the entire scaffold and are populating the open pore space. Three-dimensional rendering of the cell-seeded scaffolds showed that cells were distributed evenly on the surface and in the inner parts, forming the exact structure of the scaffolds (Figure 5C).

### Extracellular Matrix Calcification

**FIGURE 6:**
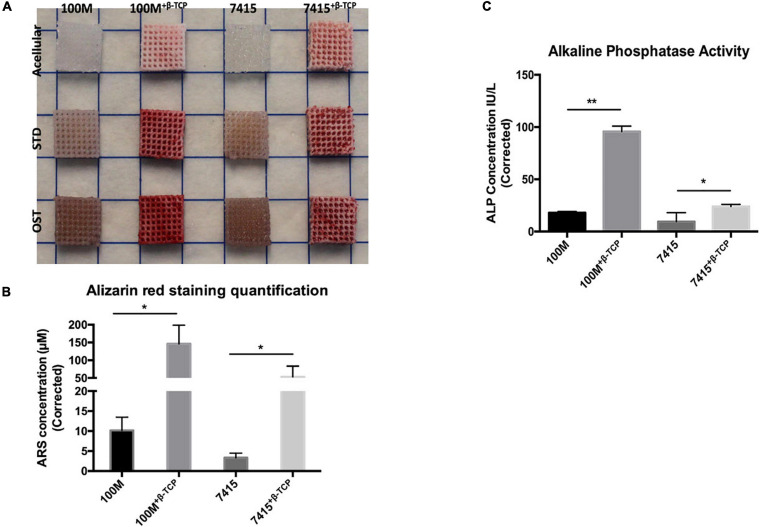
Mineralized matrix deposition. **(A)** Representative image of fixed scaffolds seeded with BM-MSC stained with Alizarin Red-S stain after 21 days culture (*n* = 3 independent experiments). Marker represent 10 × 10 mm squares dimensions. **(B)** Stain quantification using Alizarin Red osteogenesis kit representing measurements of ARS concentrations of osteogenic culture normalized to standard culture (*n* = 3), error bars represent ±SD, and *P-value < 0.05. **(C)** Alkaline phosphatase activity assay measurements represent ALP concentrations of osteogenic culture normalized to standard culture (*n* = 3), error bars represent ±SD, and *P-value < 0.05, **P-value < 0.001.

ECM calcification was visualized and quantified by Alizarin Red staining (Figures 6A,B). The visualized staining results coincided well with the quantification. After 21 days, ECM calcification on 100M^+β^^-*TCP*^ and 7415^+β^^-*TCP*^ were significantly enhanced (*P*-value < 0.05) compared to the same groups lacking β-TCP, revealing that β-TCP incorporation stimulates ECM mineralization of human BM-MSC (100M: 10.17 μM ± 3.29 SD, 100M^+β^^-*TCP*^: 146.61 μM ± 52.10 SD, 7415: 3.33 μM ± 1.12 SD, 7415^+β^^-*TCP*^: 53.31 μM ± 30.40 SD). Furthermore, 100M^+β^^-*TCP*^ demonstrated a significantly higher ALP activity (*P* < 0.0001) than that of other materials. Data presented in Figure 6C represent the cell-seeded scaffolds cultured with osteogenic media normalized to scaffolds cultured in STD. The 100M group showed a statistically significant increase with the addition of β-TCP (100M^+β^^-*TCP*^: 95.68 ± 5.31, 100M: 16.09 ± 3.0, *P* < 0.005). The 7415 group also showed a significant increase in ALP activity (7415^+β^^-*TCP*^: 23.99 ± 1.96, 7415: 17.96 ± 1.27), although the overall activity was much lower than for the 100M group. These results are in line with detection of calcified matrix on the scaffolds.

### Biosafety Analysis

**FIGURE 7:**
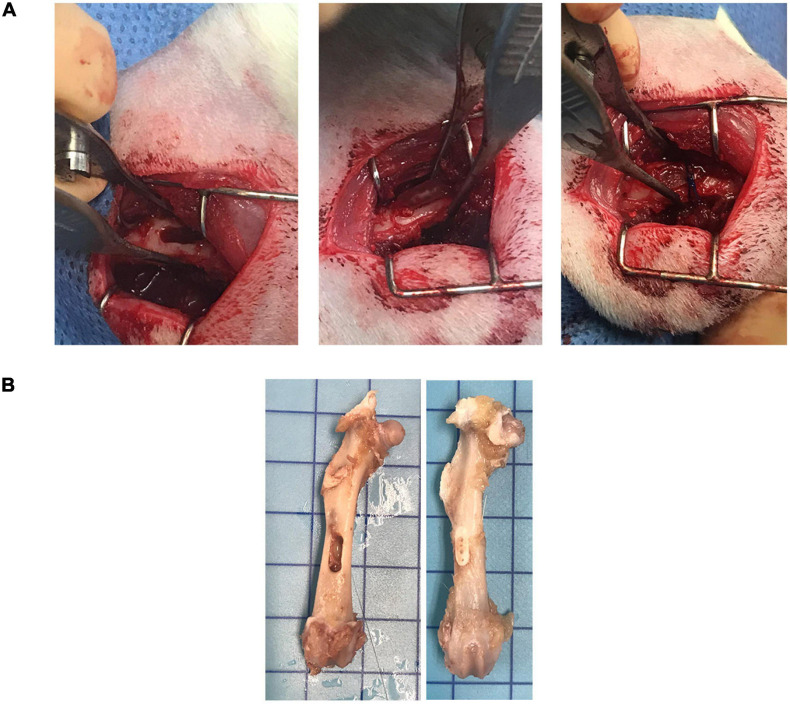
Surgical procedure. **(A)** A total of 6 mm × 2 mm window defect was created by connecting three circular defects using 1.9 drill bur. Scaffold was implanted inside the defect and suture was applied to stabilize the scaffold. **(B)** Representative image of pre-implanted and 6 weeks post-implanted femur with the marker representing 10 mm × 10 mm square.

As the 100M scaffolds showed superior performance *in vitro*, their biosafety was evaluated *in vivo*. Femoral window defects were surgically generated in rats, before implantation of 100M and 100M^+β^^-*TCP*^ scaffolds (Figure 7). Blood samples were collected from rats throughout the procedure for biosafety analysis. Blood collection pre and 6 weeks post implantation results displayed no change and values obtained were within the normal range of complete blood count (CBC), liver function test (LVT), and renal function test (RFT) (Table 2). Rats were assessed daily for signs of pain, disability, irritability, and distress. According to the observers, none of the rats showed such signs (even the ones with fractured femora).

**TABLE 2:** Hematology and biochemistry blood analysis.

			100 M		100 M^+^^β^^–TCP^	
Laboratory test	Unit	Normal range	Pre-implantation value	Post-implantation value	Pre-implantation value	Post-implantation value
RBC	10″6/ul	7.27–9.65	9.75	8.93	9.47	9.35
WBC	10″3/ul	5.5–11	11.6	12.9	12.4	11.89
Platelets	10″3/ul	300–750	1038	1043	975	955
Hemoglobin	g/dl	13.7–17.6	15.6	15.6	15.84	15.82
Hematocrit	%	41–50	45.8	49.71	45.66	43.81
MCV	fl	57–68	55	52	52	55
MCH	pg	19–22	27.2	26.7	20.7	21.1
MCHC	g/dl	32.9–37.5	32.4	34.7	33.3	37.1
Total Protein	g/L	53–69	66	64	51	53
Albumin	g/L	38–48	38	41	45	45
BUN	mmol/L	3.2–7.5	6.8	6.4	7.1	7.4
Cr	μmol/L	50–73	24	31	56	53
ALT	U/L	20–61	40	34	58	55
AST	U/L	39–111	112	89	174	161
ALP	U/L	16–302	182	201	150	174
GGT	U/L	0–6	<10	<10	<10	<10

*Blood collection analysis showing values CBC, RFT, and LVT of representative rats implanted with 100M or 100M^+β^^-*TCP*^ scaffolds pre- and post-implantation, normal range values were gathered from Charles River Laboratories data sheet.*

### μCT Analysis of Bone Formation in Femur Defects With Implanted Scaffolds

**FIGURE 8:**
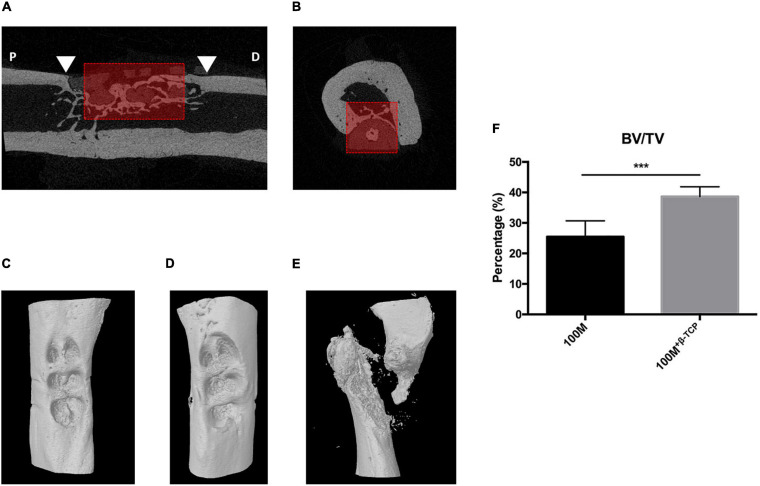
μCT ROI selection. A ROI measuring 4 mm long × 2 mm wide × 2 mm deep was delineated in the middle of the scaffold implant area (red box). Mineralized tissue and the scaffold were segmented by using different threshold values. Mineralized tissue content was quantified in the ROI. Arrowheads in **(A)** represent the edge of the defect, P: proximal, D: distal, dashed line represents the trans-axial section in **(B)**. **(C)** Reconstructed image representing defect area after 6 weeks implantation with 100M^+β^^-*TCP*^. **(D)** Reconstructed image of the defect area after 6 weeks of 100M implantation. **(E)** Reconstructed image of fractured femur implanted with 100M scaffold. **(F)** μCT quantitative analysis of (BV/TV) (*n* = 8), error bars represent ±SD, and ***P-value < 0.001.

Micro-computed tomography reconstructed images demonstrated full closure of defects in femurs implanted with 100M^+^^β^ ^–*TCP*^ at 6 weeks post implantation with difference in bone formation rate, thickness and integration. Half of the animals implanted with 100M presented with fractures at the implantation site and were excluded from μCT quantifications. Quantitative μCT analysis showed significantly increase (BV/TV) parameters in the femora implanted with 100M^+^^β^ ^–*TCP*^ scaffolds compared with femora implanted with 100M. This increase in bone mass was reflected by significantly higher trabeculae number and thickness (Tb.N and Tb.Th, respectively) that exhibited less separation (Tb.Sp), less porosity (Po.Tot), and more osteocyte lacunae (Po.N.cl) (Figure 8 and Table 3).

**TABLE 3:** μCT quantification.

Value	100M	100M^+β^^-*TCP*^	*P*-value
BV	4.11 ± 0.84	6.85 ± 0.53	0.04
BV/TV	25.46 ± 5.22	38.65 ± 3.21	0.0006
Tb.Th	165.06 ± 25.49	225.82 ± 25.78	0.07
Tb.Sp	747.12 ± 142.79	460.05 ± 75	0.04
Tb.N	1.53 ± 0.14	2.10 ± 0.35	0.02
Po.N.cl	1691.25 ± 326.51	2796.14 ± 608.33	0.05
Po.Tot	71.53 ± 5.22	60.02 ± 3.89	0.07
Conn.Dn	2.38 ± 0.51	11.70 ± 0.54	0.01

*BV, bone volume; BV/TV, bone volume/tissue volume; Tb.N, numbers of trabeculae; Tb.Th, thickness of trabeculae; Tb.Sp, trabeculae separation; Po.Tot, total porosity, Po.N.cl, number of osteocyte lacunae.*

### Histological Analysis

**FIGURE 9:**
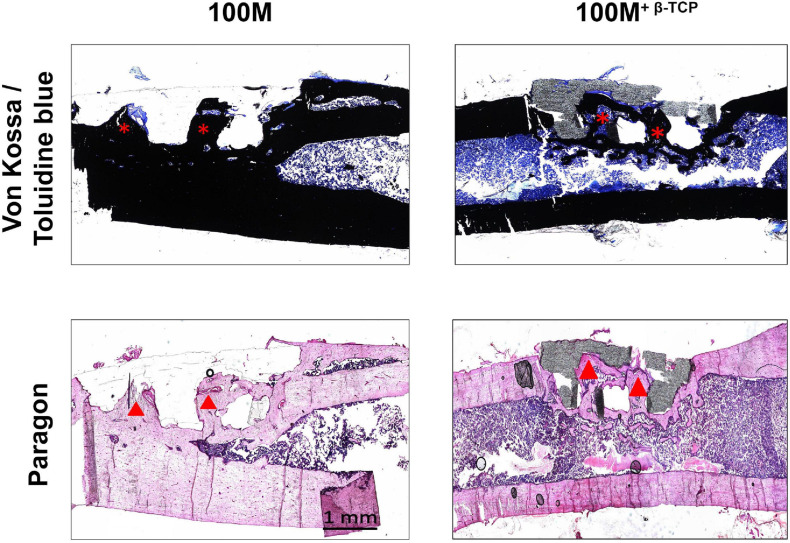
Histological evaluation of implanted scaffolds. Histological sections of un-decalcified bone stained with Von Kossa and toluidine blue (VK/TB) to distinguish mineralized (black) from un-mineralized tissue (blue) were prepared (top panels). Representative mid-sagittal images show mineralized tissue surrounding the implants and inside of their pores (asterisks). In line with the micro-CT findings, a greater quantity of mineralized tissue was observed surrounding the 100M^+β^^-*TCP*^ implanted scaffolds. Paragon staining (lower panels) showed significantly higher osteoid content in the interface between mineralized tissue and scaffold (dark pink line, arrowheads). Images were obtained at 2.5× magnification and are representative of *N* = 8 100M and *N* = 16 100M^+β^^-*TCP*^ scaffolds at 6 weeks post-implantation.

**FIGURE 10:**
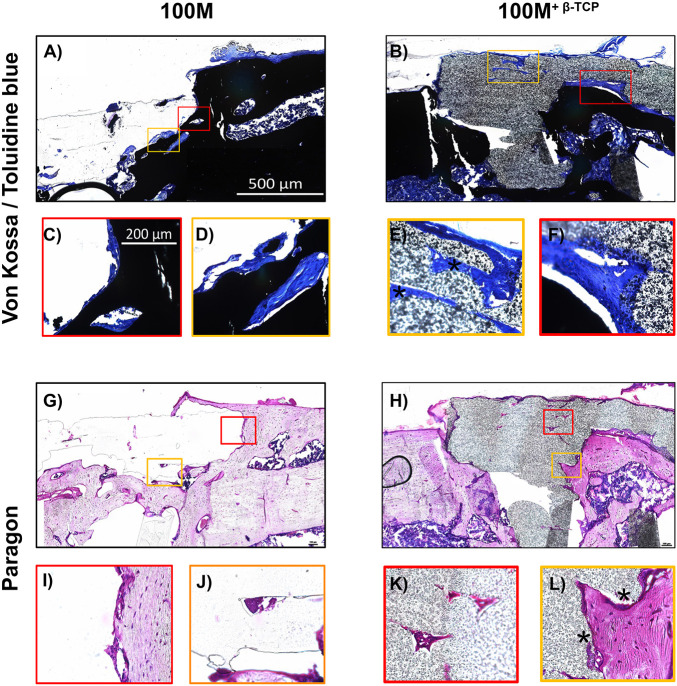
High magnification evaluation of implanted scaffolds. High magnification images of Von Kossa/Toluidine blue **(A–F)** and paragon staining **(G–L)** confirmed an increase in osteoid content in the 100M^+β^^-*TCP*^ implanted scaffolds in the interface between mineralized tissue and scaffold. Osteoid and structures resembling vascular channels were even found inside of the 100M^+β^^-*TCP*^ implant **(E,L)**, as well as a periosteal-like structure covering it (asterisk). Taken together, these findings strongly suggest that the 100M^+β^^-*TCP*^ implant is invaded by cells that are able to proliferate inside of its filaments, thereby promoting an adequate environment to foster tissue regeneration. The granular appearance of the scaffold is due to its β-TCP content. In contrast, limited tissue ingrowth and neither a periosteal-like membrane or blood vessels were found in 100M scaffolds. Images were obtained at 10× **(A,B,G,H)** or 40× magnification **(C–F,I–L)** and are representative of *N* = 8 100M and *N* = 16 100M^+β^^-*TCP*^ scaffolds at 6 weeks post-implantation.

To assess quality of the new bone formation within the scaffolds of the window defects, non-decalcified 6-μm sections of femora with 100M and 100M^+β^^-*TCP*^ implanted scaffolds were stained with Von Kossa/Toluidine blue and paragon stains. As indicated in the images in Figure 9, both scaffolds showed signs of mineralized tissue formation (dark black Von Kossa positive areas) within the pores of the scaffolds (asterisks). Paragon staining verified the presence of osteoid structures within the pores of scaffolds (arrow heads). However, visualization at higher magnification indicated that 100M^+β^^-*TCP*^ scaffolds showed much more bone deposition in the pores as well as within the scaffold itself, compared to the 100M scaffolds (Figure 10).

## Discussion

Complex trauma reconstruction, tumor resections, orthopedic infections, and spinal fusion surgeries remain among the most difficult bone repair scenarios in orthopedic surgery. Precise preoperative planning is critical especially when encountering different clinical scenarios such as treating unique, large bone defects or reconstruction post tumor or degenerate tissue resection which can require diverse surgical techniques and multiple surgical procedures (Oh et al., 2008). Treating a defected bone can be achieved by insertion of a metallic internal fixation device such as a plate and screws, metallic cage or by using an external fixator (Cattaneo et al., 1992). However, a bone transplant is usually required to bridge or to fill the defects together with these fixation procedures (Green and Dlabal, 1983; Kocaoglu et al., 2006). Notwithstanding the recent advancements and promising biomaterial developments in the tissue engineering field over the past few years, the ideal bone graft substitute and spacer materials have not yet been identified. It is therefore essential to investigate an effective and practical material and model to replace/improve the standard options and achieve functional structural integration of the engineered tissue. Recently, biomaterials in 3D printing have been attracting more attention for bone filling and regeneration (Charbonnier et al., 2019; Distler et al., 2020; Korn et al., 2020). The ideal biomaterial for graft replacement and bone healing must be biodegradable to be gradually substituted by newly formed tissue. It also needs to be bioactive to support and enhance the growth and differentiation of relevant cell populations, facilitate their interactions, promote matrix production, and characterized by suitable mechanical properties close to the native bone to provide stability and help to restore the structural alignment (Qu et al., 2015). It is very important to highlight that the role of tissue engineering is not confined to develop new tissue or replace defective ones; tissue engineering advances are widely involved in many aspects of regenerating, restoring, and repairing and also can be involved in improving current standard techniques allowing for better outcomes. Bone graft alternative materials ideally fit the description of planned surgeries that requires plenty of time and delineation. Yet, with the advancement and technological capabilities especially in the fields of 3D printing and scanning, it is reasonable to think that we will have 3D-printers connected to the emergency department system or in the operating rooms that could translate rapid 3D scans in emergent trauma situations.

The process of spinal bone healing, whether in a fracture, bone excision or fusion circumstances, is controlled first through an inflammatory phase and osteoid formation. It is then followed by processes of bone deposition by osteoblasts, bone resorption by osteoclasts, and finally remodeling (Marsell and Einhorn, 2011). Bone grafts are used to facilitate healing or arthrodesis (such as spinal fusion) and the physical, structural and chemical properties of grafts will directly affect the graft osteointegration (physical interaction of implant/bone and interpenetration) (Shah et al., 2019). Autologous bone grafts, allografts are standard approaches for various bone defects, and they promote bone repair. Autografts and allografts, however, may have complications such as pain associated with harvesting procedures, increased morbidity with limited quantities, infection, and adverse immune effects have favored the development of synthetic bone-like alternatives (Wuisman and Smit, 2006; Shalash et al., 2013). Ceramics are bioactive bone substitutes which are typically applied to non-load bearing defects, and also promote bone repair. Metallic implants, polyetheretherketone (PEEK) devices and medical acrylic cements are synthetic substitutes typically used to fill/stabilize bone defects or prosthetics where load-bearing is applicable. Limitations of the current treatment options are (1) synthetic substitutes like PMMA comprised of fixed structures often lack osteoconductive/inductive properties and/or (2) bioactive cement pastes (calcium phosphates) promote bone regeneration yet do not possess high structural mechanical strength on their own (Ahangar et al., 2019a). These limitations are particularly concerning in the context of tumor resection and trauma reconstructions in the spine for example. A bone substitute which matches bone strength and promotes bone repair would be an ideal candidate.

In this study, we used a low-cost fused deposition 3D-printer to fabricate 3D-printed constructs using our previously described design with two novel commercial materials composed of pure lactide (100M) and a blend of copolymers (lactide, trimethylene carbonate, and caprolactone) (7415), with and without the addition of β-TCP mineral. These materials are novel, in that they are “ready-to-print” and commercially available. Also, their biocompatibilities have not been described previously. Our scaffolds demonstrated high accuracy of fabrication. Both groups of scaffolds that lack β-TCP were easier to print, and whereas the 100M scaffolds used similar printing settings as the previously described PLA, the 7415 printing settings needed adjustment from past protocols, as they showed some elasticity. Groups containing β-TCP showed brittleness while printing; they were harder to print and needed modifications at first. 100M^±β–TCP^ showed similar mechanical properties, and both recorded considerably higher Young’s modulus values than the measurements described previously and close to the native bone which qualifies these materials for further investigation in the field of bone engineering (Misch et al., 1999; Fritsch et al., 2009; Lakatos et al., 2014). On other hand, 7415^±β–TCP^ showed significantly lower mechanical stiffness, making them perhaps more appropriate for soft tissue engineering approaches such as nerve grafting, cardiovascular, and cartilage regeneration applications (Pêgo, 2002). The difference between both materials in stiffness was expected and is derived from their composition, as 7415 contains caprolactone and TMC which lowers the modulus and tensile strength due to elasticity. Pego et al. (2003a) used a similar composition of copolymers of 1,3-trimethylene carbonate and caprolactone and copolymers of 1,3-trimethylene carbonate and D, L-lactide seeded with Schwann cells and evaluated their candidacy as nerve guides for the bridging of large peripheral nerve defects. Furthermore, they assessed the potential use of copolymers of 1,3-trimethylene carbonate and D,L-lactide in producing flexible 3D scaffolds for heart tissue engineering (Pego et al., 2003b). Rocha et al. (2014) showed that poly(trimethylene carbonate-co-ε-caprolactone) scaffolds promote axonal regeneration, prompting neurons into a regenerative phenotype. Others fabricated L-lactide-co-trimethylene carbonate into porous scaffolds by electrospinning and then seeded with human MSCs for growing artificial blood vessels (Dargaville et al., 2013).

The materials used in this study were developed by Poly-Med, Inc. using medical-grade materials familiar to regulatory bodies (such as FDA) and consistent with specifications found in American Society for Testing and Materials (ASTM) standard for semi-crystalline polylactide polymers for surgical implants (F1925-17^©^). Materials post-3D printing exhibit favorable mechanical properties and thermal stability, and they promote cell adhesion and extracellular matrix deposition. Overall, we observed that the scaffolds containing mineral showed an enhanced cytocompatibility and osteogenesis over the un-supplemented polymers. There is no doubt that the incorporation of β-TCP into polymers modified and improved their performance. Contact angle showed a significant improvement with the addition of minerals reflecting their cytocompatibility. In addition, the degradation profile of both materials significantly improved with the addition of β-TCP. Scaffolds lacking minerals were weaker and more fragile. *In vitro* observations demonstrated that all scaffolds had excellent cytocompatibility as cells grew and differentiated on all types of scaffolds. Both materials with β-TCP showed better distribution of cells over/within the entire scaffolds. It is possible that the attached cells used the mineral particles in creating pockets for cells to settle and form colonies. On the contrary, we speculate that the initial lower level of cell attachment on 100M^+β^^-*TCP*^ and 7415^+β^^-*TCP*^ scaffolds could be related to the rough surface. The presence of β-TCP particles may create a precipitous surface that is less conducive to adhesion but more suitable for cell growth, similar to another report (Li et al., 2016). Guo et al. (2019) also described a similar observation regarding cell attachments with the addition of calcium phosphate silicate ceramic to their poly(L-lactic acid) tendon to bone films. Furthermore, scaffolds containing β-TCP showed a significantly improved calcified matrix production and ALP activity compared to the standard scaffolds lacking β-TCP. Of all, the 100M^+β^^-*TCP*^ scaffolds showed optimal cell activity, as demonstrated by the highest calcified neo-matrix deposition and bone-like matrix. An encouraging finding was the spontaneous osteogenic differentiation of human MSC on the 100M^+β^^-*TCP*^ and 7415^+β^^-*TCP*^ materials which is described for the first time for these materials. One important improvement aspect of these novel materials is the purity of the lactide component that is associated with less adverse reactions described in earlier studies (Bergsma, 1995) and a slow degradation rate, thus the temporal concentration of degradation products as well.

Implantable versions of our scaffolds displayed a high safety level profile when evaluated *in vivo* as evidenced by normal values of blood panels and no signs of local or systemic inflammation at the evaluated time point. We speculate that scaffolds recruited host-resident stem cells in the adjacent bone marrow, where the addition of β-TCP demonstrated augmented osteoconduction as evidenced by enhanced closure of defects and statistically significant increase in the μCT parameters compared to implants lacking the additive minerals. Furthermore, scaffolds have shown favorable interactions with bone and no obvious absorption of the polymers were detected during the period of implantation. Materials seemed to follow the known degradation pattern typical of biodegradable polyesters *in vivo* (Bos et al., 1991; Grayson et al., 2004). Surprisingly, half of the group implanted with 100M showed evidence of fracture at the site of the defect. These fractures were unlikely due to human error or intra-operative complication as post-operative radiographs confirmed implant position and intact cortex. Furthermore, animals did not show any signs of pain or disability during follow-up period. Additionally, the thin fibrous capsule around the fracture site suggested that fractures occurred in between 4 and 6 weeks post-implantation. We speculate that β-TCP enhanced healing by attracting more cells to the defect site, promoting osteogenesis and providing structural support. However, a larger sample size and perhaps a larger animal model with segmental defects would demonstrate additional valuable data, and further analysis such as immunohistochemical are required for further assessment.

Data presented in the current study on these innovative biomaterials for bone graft substitution represents a continuation of our previous work emphasizing the use of low-cost 3D printing for high resolution translational scaffolds (Fairag et al., 2019). It provides preclinical proof of concept that simple 3D-printing using FDA-GRAS (generally regarded as safe) “smart” materials designed specifically for medical implantation display superior biological performance through modulation of cell behavior and activity.

Novel biomaterials can find their way into a several applications, from biodegradable sutures to specialized surgical tools, guides, and implants. In all instances, the challenge lies in finding the ideal parameters for the specific tissue engineering application. To do so, *in vitro* biological assessment predicting the impact and performance of a material for desired application in the human body are essential along with *in vivo* experiments to define potential adverse effects and body response. Several *in vivo* studies have been conducted for different scaffolds to evaluate bone regeneration in defect models. Cipitria et al. (2013) showed promising results by using PCL scaffold with β-TCP microparticles embedded with bone morphogenetic proteins (BMPs) in critical-sized segmental defects in sheep tibiae and observed equivalent results of bone bridging to autograft within 3 months. Although they used their model as a reservoir for BMP release, they have proven that with optimization of scaffold design, 3D printed scaffolds showed clinical promise (Cipitria et al., 2013). Inzana et al. (2014) fabricated calcium phosphate Inkjet-based 3D printed scaffolds and implanted them into mouse femora. They observed new bone growth within the implants similar to allografts after 9 weeks (Inzana et al., 2014). Similar results were observed in other studies of 3D printed implant in non-weight bearing bones (Igawa et al., 2006; Yoon et al., 2007; Tamimi et al., 2009). Although most of these studies successfully proved the principle of employment of scaffolds in bone healing, there is still a scarcity of a reliable standardized small-size animal model. Different research groups have developed various fracture model designs, yet they depend to a certain extent on several factors such as breed, age, sex, or weight of the animals. A wide range of defects (between 2 and 10 mm) have been described in the literature (Drosse et al., 2008). Thus, a 6-mm defect size in male rats here was chosen representing the average reliable length of critical sized defect in a weight-bearing bone. However, the cortical window defect preserved the stability of the bone while also exposing the implants to both biological and mechanical factors allowing healing processes without the need for instrumentation. This simple *in vivo* model serves the main objectives in this study which were to evaluate the safety and potential for bone repair by these implantable scaffolds.

The materials used here could also be promising candidates as alternatives to metallic cages used in spinal fusion surgeries as space fillers. Analogous principles and physiological phases of incorporating a bone graft or an implant into a disc space in a spinal fusion surgery take place in the healing process of long bones (Boden et al., 1995). In fact, van Dijk et al. (2002a) evaluated the potential benefits and long-term performance of bioabsorbable poly-L-lactic acid (PLLA) cages as an alternative to titanium cages in a goat spinal fusion model. The authors found significantly (*P* = 0.04) higher rate of lumbar interbody fusion associated with PLLA cages than those with titanium cages of the same design after 6 months. PLLA cages demonstrated absorption and maintenance of interbody fusion around 1 year, with complete bone remodeling within PLLA after 2 years (van Dijk et al., 2002a). Furthermore, they showed that at the 4-year follow-up (0–1%), of the original PLLA could be observed (van Dijk et al., 2005). However, due to the difficulty and delicacy of executing spinal fusion surgery *in vivo*, only a few of animal models are available (Wang et al., 2003; Zou et al., 2010; Dang et al., 2018). Rats are commonly used as *in vivo* spinal fusion models. Due to their considerably small size, rats have usually been used for non-instrumented fusions. Others have tested their advances by implanting them into long bones simulating spinal fusion.

The purpose of evaluating potential for bone repair in this preliminary *in vivo* experiment was to identify whether 100M or 100M^+β^^-*TCP*^ present appropriate materials for further *in vivo* investigation in critical sized defects at 8- and 12-weeks post-implantation. Demonstration of 100M^+β^^-*TCP*^ as a bone graft alternative will require more clinically relevant experimentation such as implantation into a full segmental critical defect with instrumental fixation and a lengthy follow-up period compared to a standard graft material. It has become clear from this study and the available literature that biodegradable materials represent promising alternatives to be used as bone graft or interbody spacers in reconstruction and fusion surgeries by providing initial stability for bone healing. This is followed by gradual resorption of the material and steadily shifting the load to the healing bone and the void filled with bone. However, the optimal type of material with the appropriate biological, physical, and structural properties is still undiscovered.

## Conclusion

In conclusion, the current study establishes a proof of concept that low-cost 3D-printing using novel materials that are consistent with FDA guidelines and ASTM standards can be promising candidates for bone graft alternatives in the field of orthopedic and reconstructive surgery. 100M^+β^^-*TCP*^ scaffolds particularly showed excellent mechanical properties, biological characterization, and supported bone matrix formation. The enhanced osteoconductive properties that β-TCP adds to the materials has an important clinical translation. The attraction of more cells along with supporting proliferation and calcified matrix production, with a balanced degradation profile which allows new bone formation and material resorption, represents a significant insight in developing novel approaches to improve current surgical techniques. Use of these innovative materials through additive manufacturing is a novel tissue engineering approach to improve standard surgical outcomes that is only beginning to develop. Further analyses are required with appropriate evaluation in a larger critical-sized defect animal model with long-term follow-up are needed and substantiated based on the present evaluation.

## Data Availability Statement

The raw data supporting the conclusions of this article will be made available by the authors, without undue reservation.

## Ethics Statement

The animal study was reviewed and approved by RI-MUHC 2018-8038.

## Author Contributions

Conceptualization was done by RF, DR, MW, and LH. Experimental designing, reviewing, and editing were done by DR and LH. Experimentation and data analysis were carried out by RF and LL. μCT scans were done by JR-G. Polymers characterization was analyzed by MT and BG. Resources and material were provided by LH. Results’ interpretation was done by RF, LL, and LH. The original manuscript was written by RF. Project supervision was done by DR, MW, and LH. All authors approved the final version of the article for submission.

## Conflict of Interest

MT and BG are employees of Poly-Med, Inc. The remaining authors declare that the research was conducted in the absence of any commercial or financial relationships that could be construed as a potential conflict of interest.

## Abbreviations

3D: three-dimensional
PLA: polylactic acid
PCL: polycaprolactone
100M: pure lactide polymer
7415: copolymeric material
*PEEK*: polyetheretherketone
PLGA: poly (lactic-co-glycolic acid)
β-TCP: beta-tricalcium phosphate
DMEM: Dulbecco’s Modified Eagle Medium
STD: standard culture media
OST: osteogenic culture media
GuHCl: guanidine hydrochloride
XPS: X-ray photoelectron spectroscopy
PFA: buffered paraformaldehyde solution
ALP: alkaline phosphatase
μ CT: micro-computed tomography
ROI: region of interest
BV/TV: bone quantity
Tb.N: number of trabeculae
Tb.Th: thickness of trabeculae
Tb.Sp: separation of trabeculae
Conn.Dn: connective density
Po.Tot: total porosity
Po.N.cl: number of osteocyte lacunae
PMMA: polymethylmethacrylate
MSS: multiple stain solution
SD: standard deviation
ANOVA: analysis of variance
DSC: differential scanning calorimetry
T_*g*_: glass transition temperature
T_*c*_: peak of the crystallization endotherm
Δ H_*c*_: heat of crystallization
T_*m*_: peak of the melting endotherm
Δ H_*f*_: heat of fusion
GPC: gel permeation chromatography
M_*n*_: number average molecular weight
M_*w*_: weight average molecular weight
PDI: polydispersity index
IV: inherent viscosity
GC: gas chromatographic technique
SEMs: scanning electron microscopy
MSC: mesenchymal stem cells
CBC: complete blood count
LVT: liver function test
RFT: renal function test
VK/TB: Von Kossa and toluidine blue
ASTM: American Society for Testing and Materials
FDA: Food and Drug Administration
GRAS: generally recognized as safe
BMPs: bone morphogenetic proteins
PLLA: poly-L-lactic acid.
